# Characterization of Encapsulated Berberine in Yeast Cells of *Saccharomyces cerevisiae*

**Published:** 2015

**Authors:** Roshanak Salari, Omid Rajabi, Zahra Khashyarmanesh, Mohsen Fathi Najafi, BiBi Sedigheh Fazly Bazzaz

**Affiliations:** a *Department of Drug and Food Control, School of Pharmacy, Mashhad University of Medical Sciences. *; b *Targetted Drug Delivery Research Centre, School of Pharmacy, Mashhad University of Medical Sciences, Mashhad, Iran. *; c *Department of Veterinary Research and Biotechnology, Razi Vaccine and Serum Research Institute, Mashhad, Iran. *; d *Biotechnology Research Centre, School of Pharmacy, Mashhad University of Medical Sciences, Mashhad, Iran.*

**Keywords:** Berberine, kinetic, MIC, microencapsulation, yeast cells

## Abstract

Berberine was loaded in yeast cells of *Saccharomyces cerevisiae*as a novel pharmaceutical carrier to improve the treatment ofmany diseases. The yeast-encapsulated active materialsshowedhigh stability and bioavailability due to the enhanced solubility and sustained releasing. In this study, different characteristics of prepared berberine loaded yeast cells (loading capacity, release kinetic order, MIC and stability) were evaluatedby different analytical methods (fluorescence spectroscopy, HPLC and SEM).The loading capacity was about 78% ± 0.6%.Berberine release patterns of microcapsules happened in two different stages and followed by zero and first-order kinetic,respectively. About 99% of all active material released during 34 h. MIC was improved by berberine loaded microcapsules in comparison withberberine powder. The microcapsules were completely stable. Berberine loaded *Sac. Cerevisiae *could be considered as a favorite sustained release drug delivery system. The yeast would be applied as an efficient carrier to improve various properties of different active materials.

## Introduction

In every encapsulation process, physical characteristics of the product for example encapsulation efficiency, the release kinetic of active material,etc should be determined and optimized for better drug encapsulation (1). Kineticsis the study of rates of different reactions. Itprovides information about the reaction's mechanism by mentioning mathematical models which describe the characteristics of a chemical reaction. Kinetics means how various conditions can affect the speed of a chemical reaction. Kinetic studies for pharmaceutical dosage forms in medicine is very important because the final results help us to know that how many times a drug should be prescribed a day. Patients are more familiar with such medicineswhich are prescribed once a day (2).

Microencapsulation is a process that is applied a lot in pharmaceuticals, cosmetic and food industries due to its extension of shelf-life, protection against oxidation and control release of active component (3).Microencapsulation nowadays developed in many fields of sciences and different drug and food industries. It is known as a process in which small active compounds are surrounded by another material, which is called coat or carrier. Encapsulation causes stabilization to the active materials since the wall material acts as a physical barrier to protect the active compound. Besides, the material can be released in a controlled way in product applications (4).

The structure of yeast cell wall made it an excellent encapsulating material and its natural properties caused many advantages over other microencapsulation carriers (5).Yeast-cell-based microencapsulation process has been applied in the encapsulation of different materials such as essential oils and flavors (6). Baker’s yeast (*Saccharomycescerevisiae*) has been proved as anappropriate host for the development of a new kind of drug delivery system (7).

*Sac.cerevisiae* yeast cellscan be regarded as food-grade and low cost food materials (8). Their phospholipidmembranes could behave as liposomes and have been used for the encapsulation ofdifferent molecules with different lipophilicity (9).The yeast cell wall composed of beta glucan network and a small amount of chitin with a mannoprotein layer which made it more beneficial comparing to other carriers. The wall allows molecules to diffuse conveniently (10).

Berberine has many pharmacological properties. Several pharmacological studies have indicated the cardiovascular effects of berberineagainst ischemia induced by ventricular tachyarrhythmia.It is used to treat cardiac contractility and decreasing blood pressure (11, 12).Berberine could improve the damaged heart function of CHF rats (13),has high capacity in antiplatelet aggregation in patients (14) and itshows immunosuppressive influences (15).Recent reportshowed berberine could be a good candidate for further studies as a new anticancer drug in the treatment of human breast cancer (16).

Many studies have been shown antiinflammatory activity for *Berberis vulgaris* and its most alkaloid, berberine, but the exact mechanism is unknown (17, 18).*B. vulgaris* fruit (barberry) wasadministered for its sedative effect in traditional medicine (19).It is reported that the intake of 3 g/d *B. vulgaris* fruit extract for 3 months may have beneficial effects on different factors in type 2 diabetic patients,such as lipoproteins, apoproteins, glycemic control and TAC (20). Berberine has usually been used as an antihyperglycemicmedicine in China (21). Berberine can be used as an expectorant according to its ability to increase mucin release (22). Berberine has been known to cure diarrhea in different population (23). Berberine low solubility and its instability to environmental conditions are the major problems that prevent its consumption in various industries (24, 25).

In this work, the physical characteristics of berberine loaded microcapsules were studied.

## Experimental

*Materials*

*Saccharomyces cerevisiae*lyophilized powder was purchased from Industrial Research Center (PTCC No. 5269), Tehran, Iran. Berberine hydrochloride was obtained from China (XI ANRongsheng Biotechnology CO., LTD (. Double distilled water was prepared to carry out the experiments.Tryotone soya broth (TSB) culture medium was purchased from Himedia, India. Merck brand of tetrazolium salt was used in the experiments.

*Preparation of microcapsules*

Microcapsulation was performed with some modification according to the method by Paramera, *et al .* (8). The freeze dried yeast cells (7 mg) were suspended in a flasks containing 10 ml berberinein water solution (275 µl/ml) at 45ºC. The flasks were stirred at 200 rpm for 72 h and then centrifuged (7000 rpm, 15 min). The precipitants were washed three times to remove the free and excess berberine. Then the microcapsules which loaded with berberine were freeze dried.

*Preparation of standard solutions to plot the standard curve*

Berberine standard solutions were prepared by dissolving different amounts of berberin in double distilled water to obtain five standard concentrations. The concentrations were 500, 400, 300, 250, 125, 62.5 and 31.25 µl/ml. The fluorescence emissions of these concentrations were measured and the standard curve was plotted.

*Determination of loading capacity*

To define the encapsulation efficiency, 7 mgberberine loaded microcapsules was dissolved in 10 ml double distilled water. This suspension was stirred (200 rpm) for 48 h. The final supernatant was centrifuged and the fluorescence emission (Shimadzu spectrophotometer RF-540) was determined. Based on the standard curve, the concentration of loaded berberine was determined.

*Release kinetic studies*

Microcapsules (7 mg) (equal to 215 µg/ml pure berberine) were suspended in 10ml of water in a flask (kinetic flask). The flask was put on a stirrer (100 rpm) for 48 h. The samples (100µl) were centrifuged at predetermined time points and the supernatant was separated. The precipitatedmicrocapsules were suspended in 100 µl distilled water and returned back into the flask (26). The supernatants were then analyzed by fluorescencemethod.

In fluorescence spectrophotometry method, measurements were carried out in 520 nm (Ex: 375 nm).According to the standard curve, the concentration of free berberinein each sample was determined. Due to higher sensitivity of fluorescence method, mathematical calculations to estimate berberine release order carried out based on fluorescence data. Matlab mathematical software was used to define the equations of all curves.

Effect of pH on berberine release process was studied by introducing 0.5 ml 0.01 M HClto the kinetic flask. The experiment was carried out in the waymentioned above.

*Antimicrobial activity measurement*

Minimum inhibitory concentration (MIC) was evaluated for berberine as an active material,berberine loaded microcapsules and physical mixture of berberine and yeast cells (5 mg). Three organisms (*Staphylococcus aureus*(PTCC 1337)*, Pseudomonas aeruginosa*(PTCC 1707) and*Esherichia coli*(PTCC 1330))were used to estimate the MICs. 20 µlof 10^6^ suspensions of each microorganism was added to 200 µl of three antimicrobial agents (berberine, berberine loaded microcapsules and physical mixture of berberine and yeast cells) in differentpure berberine concentrations (500,400,300,200,100, 75,50, 37.5 µg/ml) in separate wells of 96 wellmicroplate. The plate was incubated for 24 h. After incubation, 50 µl of tetrazolium salt (C_19_H_15_C_l_N_4_) solution (5mg/ml) was added to each well. Then incubation was done for 45 min. The well before the one that the color of its content became red, showed us MIC.

To define the Minimum bactericidal concentrations (MBC_S_), 20 µl of all concentrations higher than MIC which showed no microorganisms growth, were added to 200 µl TSB culture medium in separate wells. The microplate was incubated for 24 h. Tetrazolium saltsolution was used to distinguish the live microorganisms that they were not killed, and in fact only their growth was inhibited by the active material. The well before the one that its red color was appeared, indicated MBC concentration. These experiments were repeated for three times.

*Stability of microcapsules*

The stability of microcapsule powder was studied according to ICH time points (0, third month, sixth month and a year) by HPLC (Waters, 600 controllers) analytical method. The analysis was performed on a C_18 _column with phosphate buffer 0.02 mol/L (pH 5)-acetonitrile (75: 25) as mobile phase at a flow-rate of 1.0 ml/min, with UV/Visible detection at 340 nm.The pure berberine concentrations in the samples were 300µg/ml. The suspensions were centrifuged several times then supernatants (berberine solutions) were collected.

*SEM images*

Scanning electronic microscopy (Oxford Company, S-360) was used to study the cell wall morphological differences between the yeast cells and berberine loaded yeast cells. The yeast cells and microcapsules were embedded in paraffin, sectioned, de-paraffin, and sputter-coated with gold.

## Results and Discussion

In the previous study, we proved the entry of berberine inside the yeast cells by different analytic techniques (27). In this study only the physical characterization of this drug dosage form was determined.

**Figure1:**
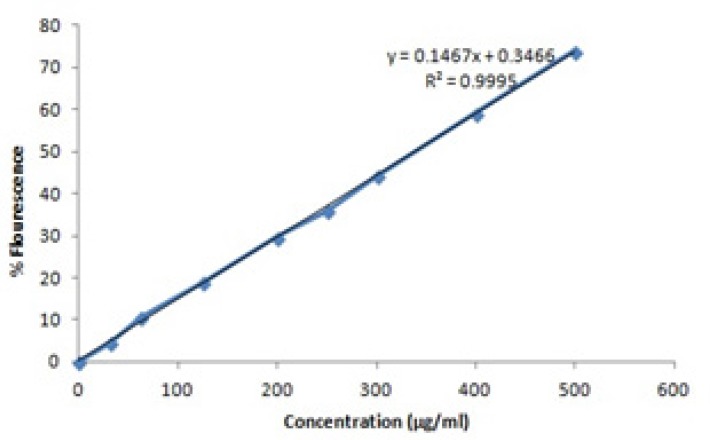
Berberine fluorescence standard curve

In order to evaluate the loading capacity and the release mechanism, fluorescence spectro photometry was applied. The standard curve was drawn based on the standard concentrations of berberine (Table 1) (Figure 1).

**Table 1:** Berberine standard concentrations data

Concentration (µg/ml)	0	31.25	62.5	125	200	250	300	400	500
% Flourescence	0	4.6	10.5	19	29.7	36	44.3	59.2	74
SD	0	0.21	0.33	1	0.2	1	0.41	0.37	1

*Loading capacity*

The flouresence emission of berberine solutionswhich obtained by degradation of microcapsules was 31.9% (concentration = 215 µg/ml). According to standard curve, the loading capacity (Equation. 1) is about 78% ± 0.6% (215/275 µg/ml × 100).

%Loading capacity = C/C0× 100

(Equation1)

In equation 1, C refers to the concentration of released berberine by degradation of microcapsules and C_0_ is the concentration of the berberine solution used for encapsulation process.Due to yeast cells availability, low cost production process and the high loading capacity, they could be applied as beneficial natural carriers to load active materials. Their unique structural properties like the beta glucan network cause the active material to release in a controlled way and it could protect the materials aginst any harmful environmental factors. In comparison with other studies, 78% loading capacity is high and completely acceptable (8).

*Release kinetic studies*

The release profile of berberine was studied and the curves equations were calculated by Matlab software.The equations which were obtained by Matlab software and showed below each curve, describe the release profile of berberine in mathematical formula.

**Figure 2:**
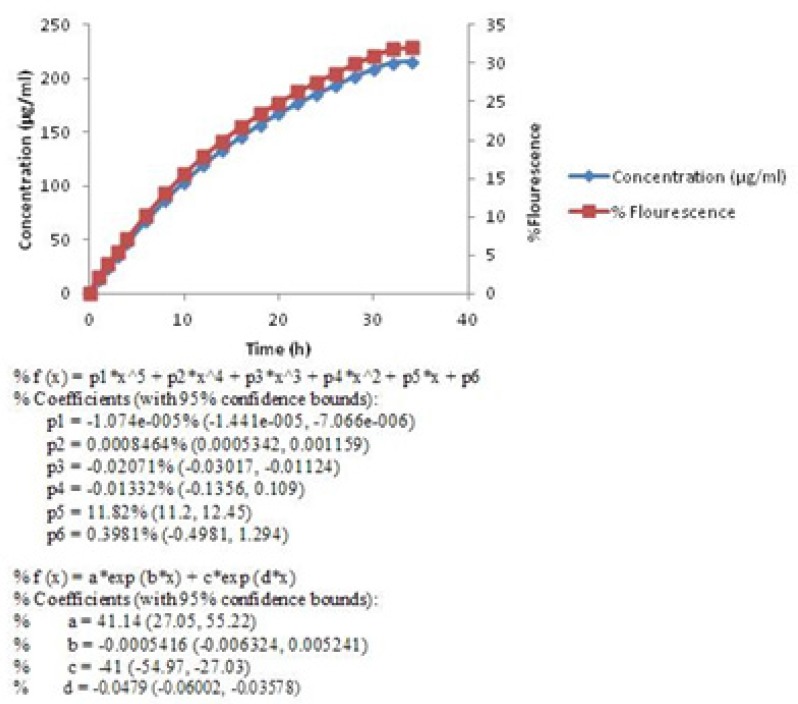
Release profile of encapsulated berberine(First equation under the curve is based on berberine%Flourescence and the second one is based on berberine concentration (µg/ml)).

**Figure 3:**
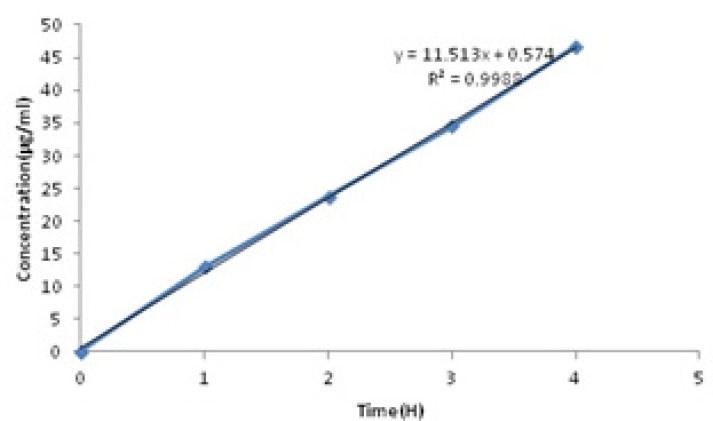
Diagram of released berberineconcentrations in the first 4 h (SD = ± 1%).

Berberine released in two stages (Figure 2). In the first stage (0-4 h), a sharp slope was observed. This sharp slope at the beginning of berberine release curve was represant of zero-order kinetic pattern which is independent of berberine concentration (28) (Figure3) (Equation 2). 

Zero order equation (Equation 2):

$Q_{t}=Q_{0}+K_{0t}$

 (Equation 2)

In Equation 2, Q_t_ is the amount of drug dissolved in time t, Q_0_ is the initial amount of drug in the solution (most times, Q_0_ = 0) and K_0_ is the zero order release constant expressed in units of concentration/time.

**Figure 4.**
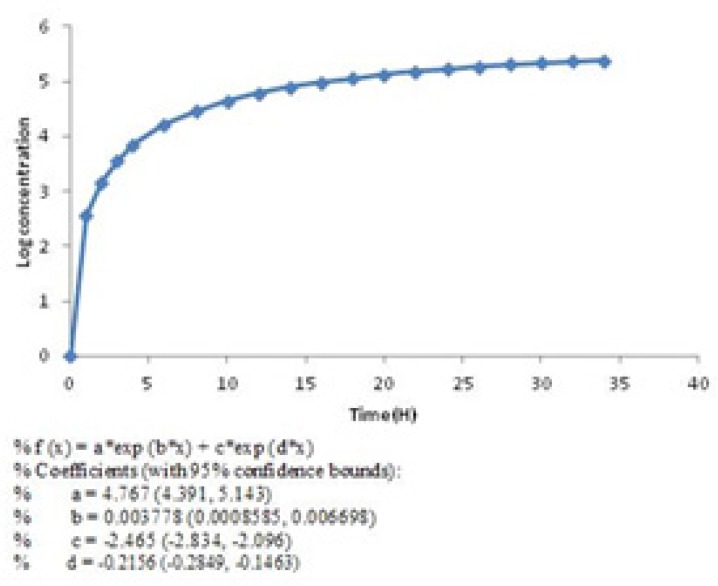
Diagram of Log berberine concentration against time (SD = ± 1%).

Then the rate of drug release was slowed down but continued until 99% of the active material (Figure 2) released in 34 h. This pattern of release followed first-order kinetic which is dependent on berberine concentrations (28). The diagram of Log concentration against time is linear about 12 h after releasing which confirms that the second stage obey first-order kinetic (Figure 4) (Equation 3).

First order equation (Equation 3):

Log C=Log C0+Kt /2.303

 (Equation 3)

In equation 3, C_0_ is the initial concentration of drug, K is the first order rate constant, and t is the time. The data obtained are plotted as log cumulative percentage of drug remaining vs. time which would yield a straight line with a slope of K/2.303.

**Figure 5:**
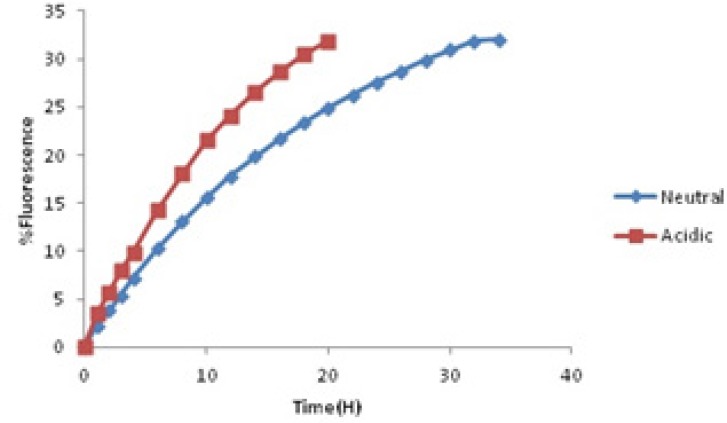
Release profile of encapsulated in acidic (pH = 4) and neutral environment

In the first stage, berberine release pattern obeys passive diffusion mechanism and follows zero-order kinetic. Besides, the second stage follows first-order kinetic which is dependent on berberine concentration and happens by the same passive mechanism. These observations show that this kind of drug delivery system could be applied as a sustained release one (29).These microcapsules could be used as kind of natural preservative to protect foods against deterioration process outside the refrigerator for about 34 h. Effect of other parameters such as pH was evaluated too. Some foods are sour because of their acidic nature. Acidity affects the structure of yeast cells membranes and cell walls. As a result, the acids facilitated berberine release pattern and the sustained release property of yeast cells would be affected. Diagram of berberine release in acidic environment was compared with the one in neutral environment (Figure 5).

Acids could extract cell wall beta glucan network so they would cause damages to natural structure of yeast cell walls. By introducing micro capsulesto acidic environment, the time period that the whole berberine released would decrease to 20 h. It means one of the most significant properties of this carrier as the sustained release one would be damaged. But the release pattern obeys the order in neutral solution. In acidic release profile, in the first stage, zero-order release kinetic happened. Following that, releasing is dependent on first order kinetic but it was completed up to 20 h. The mathematical equations below each curve showed us the parameters that the release order is dependent on them.

*Antimicrobial activity measurement*

Berberine loaded microcapsules improve minimum inhibitory concentrations of berberine against *Staphylococcus aureus*and *Escherichia coli* from 200 to 100 µg/ml and 100 to 75 µg/ml respectivelyin comparison withother antimicrobial samples. But no difference in MIC was seen for* Pseudomonas aeruginosa *(300 µg/ml). Physical mixture of berberine and yeasts cells showed the same MIC as berberine alone. The red color in wells of microplate is represented of the oxidation reaction between the live organism and tetrazolium salt. Encapsulation of berberine in yeast cells improves berberine penetration to the structure of microorganisms. The results show that *P.aeruginosa *might be the most resistant microorganism against berberine and its derivatives. Minimum bactericidal concentration was the same in all samples. MBCswere evaluated 300 µg/ml for *Staph.aureus*and *E.coli* and 500 µg/ml for *P.aeruginosa. *Berberine kills microorganisms by different mechanisms such as its destructive effects on DNA and RNA structures or proteins biosynthesis (30).

*Microcapsules stability studies*

**Figure 6:**
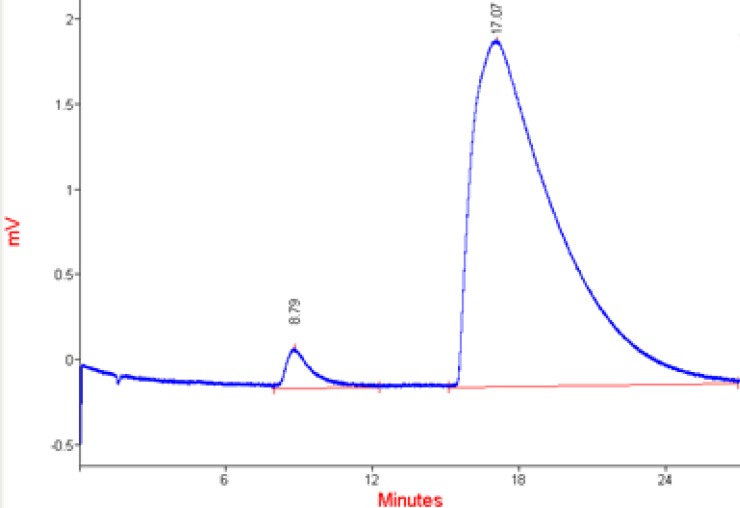
HPLC chromatogram of released berberinesolutions (300 µg/ml).

HPLC chromatogram of loaded berberine in microcapsules is shown in Figure 6.

**Table 2:** Berberine release HPLC chromatograms data.

**Time (month)**	**RT.**	**Area**	**%Area**	**Height**	**SD**
0	17.13	457.13	98.73	1.52	± 2%
3	16.46	442.27	98.68	1.61	± 1.9%
6	16.36	430.87	98.87	1.63	± 2.2%
12	15.6	425.23	98.62	1.58	±1.8%
Pure berberine	17.07	461.05	98.75	1.55	±1.9%

Berberine retention time is about 16 min. The chromatograms (Figure 6) and the data (Table 2) showed that all berberine peaks are approximately the same and the microcapsules were stable after one year of production. The small first peak is due to the impurities of berberine. All characteristics of berberine chromatograms released from microcapsules were the same as pure berberinesolution chromatogram. The stability of berberine against light, oxidation and other environmental parameters confirmed the efficiency of yeast cells as carriers.

*SEM images*

**Figure 7:**
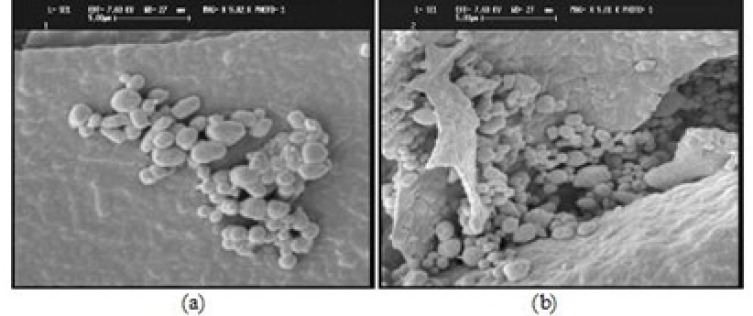
SEM images of a) yeast cells and b) berberine loaded yeast cells

The SEM images of yeast cells (a) and berberine loaded yeast cells (b) are shownin Figure 7.

SEM technique was applied to study the effect of encapsulation on the surface structure of yeast cells. The SEM images (Figure 7) showed no significant differences in cell wall morphological properties of the yeast cells and berberine loaded yeast cells. In fact, micro encapsulation process did not affect the cell wall organization.

Berberine is the most important alkaloid of Barberry. Its pharmacological effects play an important role in treatment of many diseases. The unfavorable properties of berberine powder are its poor water solubility and susceptibility to different environmental conditions (31). It seems reasonable to find a new carrier to improve these unfavorable properties.

Baker’s yeast (*Sac. cerevisiae*) could be introduced as a novel kind of drug delivery system. This yeast cell developed a low cost microencapsulation process. The yeast cells as carriers improve the active material shelf-life and protection against oxidation and provide control release drug dosage form. The structure of yeast cell wall and its natural properties made it a unique one over other microencapsulation carriers (32).

## Conclusion

The loading capacity, releasekinetic pattern, MIC and stability studies showed that yeast cell of *Sac.cerevisiae* as a carrier could modify unwanted properties of berberine powder and caused a sustain release and stable drug delivery system.
